# Bidirectional band-selective magnetization transfer along the protein backbone doubles the information content of solid-state NMR correlation experiments

**DOI:** 10.1007/s10858-017-0147-0

**Published:** 2017-11-08

**Authors:** M. M. Jolly, J. A. Jarvis, M. Carravetta, M. H. Levitt, P. T. F. Williamson

**Affiliations:** 1 Centre for Biological Sciences, University of Southampton, Southampton, SO17 1BJ UK; 2 School of Chemistry, University of Southampton, Southampton, SO17 1BJ UK

**Keywords:** MIRROR, GB3, DARR, Sequential assignment, Protein NMR, Solid-state

## Abstract

Resonance assignment is the first stage towards solving the structure of a protein. This is normally achieved by the employment of separate inter and intra residue experiments. By utilising the mixed rotation and rotary recoupling (MIRROR) condition it is possible to double the information content through the efficient bidirectional transfer of magnetization from the CO to its adjacent Cα and the Cα of the subsequent amino acid. We have incorporated this into a 3D experiment, a 3D-MIRROR-NCOCA, where correlations present in the 3D spectrum permit the sequential assignment of the protein backbone from a single experiment as we have demonstrated on a microcrystalline preparation of GB3. Furthermore, the low-power requirements of the MIRROR recoupling sequence facilitate the development of a low-power 3D-NCOCA experiment. This has enabled us to realise significant reductions in acquisition times, allowing the acquisition of a single 3D-NCOCA spectrum suitable for a full backbone resonance assignment of GB3 in less than 24 h.

**Electronic supplementary material:**

The online version of this article (doi:10.1007/s10858-017-0147-0) contains supplementary material, which is available to authorized users.

## Introduction

Over the past two decades, significant advances have been made in techniques for the determination of the atomic-resolution structure and dynamics of proteins by solid-state NMR (ssNMR). This is due in part to technological advances in hardware, leading to the availability of higher magnetic fields, and faster spinning MAS probes; as well as a plethora of methodological advances and pulse sequences to determine the structure of large biopolymers.

A prerequisite for site-specific structural and dynamic studies of proteins by ssNMR is a backbone resonance assignment. While proton-detected methods of resonance assignment and structure determination are becoming increasingly more popular and less time consuming with the advent of ultrafast MAS (> 60 kHz) probes (Andreas et al. 2016; Marchetti et al. 2012; Barbet-Massin et al. 2014; Wang et al. 2015; Agarwal et al. 2014), the assignment process is still commonly achieved using ^13^C-detected experiments on uniformly ^13^C/^15^N labelled samples at rotation frequencies below 60 kHz (Theint et al. 2017; Ravotti et al. 2016; Wiegand et al. 2016; Schnell 2005, Su et al. 2014). Though other approaches are used, the most common method to assign the protein backbone with ^13^C-detected experiments is to use separate two- and three-dimensional heteronuclear NCC correlation experiments of the type NCOCA/NCOCX to obtain inter-residue correlations, and NCACO/NCACX to obtain intra-residue correlations, usually in combination with other two- and three-dimensional hetero- and homonuclear experiments (Pauli et al. 2001; Sperling et al. 2010; Schuetz et al. 2010; Higman et al. 2009). NCOCX and NCACX experiments are both typically performed with band selective polarisation transfer by cross-polarisation (CP) from ^15^N_i_ spins to either adjacent CO_i−1_ (NCOCX) or Cα_i_ (NCACX) spins, followed by a homonuclear dipolar recoupling sequence to transfer spin polarisation between local ^13^C spins. Numerous homonuclear dipolar recoupling sequences exist, each with specific features that confer advantages for particular applications or experimental conditions, as reviewed by De Paëpe (2012) and Mithu et al. (2013).

An apparent opportunity to condense the acquisition time required for sequential assignment of uniformly labelled solid proteins in this manner is to simultaneously record inter-residue (NCOCX type) and intra-residue (NCACX type) correlations in a single experiment, effectively doubling the information content of typical NCC experiments, and halving the required acquisition time. This type of bidirectional inter- and intra-residue polarisation transfer is exploited frequently in solution-state NMR experiments for protein assignment, such as HNCA (Bax and Ikura 1991) and HNCACB (Grzesiek and Bax 1992a, b). In these liquid-state experiments, polarisation on N_i_ spins is simultaneously transferred via the *J*-couplings to both the adjacent Cα_i_, and the preceding Cα_i−1_, resulting in a spectrum with both inter- and intra-residue correlations. For this reason, in favourable cases, either of these experiments can be sufficient to assign the backbone resonances of a protein in a single experiment (Grzesiek and Bax 1992a, b). This bidirectional approach is however rarely encountered in the assignment of solid proteins. This may be attributed to the fact that using the *J*-coupling to transfer polarisation in solid samples is often inefficient compared to dipolar recoupling, particularly at lower MAS rates, due to typically large inhomogeneous linewidths, and short coherence lifetimes. 3D heteronuclear correlation spectroscopy using *J*-based transfers for resonance assignment in solid proteins has been demonstrated previously, where extensive deuteration of samples can help to achieve the required linewidths and coherence lifetimes but is not essential (Linser et al. 2008; Chen et al. 2007).

In terms of using the hetero- and homonuclear dipolar recoupling methods more commonly used in ssNMR assignment methods to simultaneously record inter- and intra- residue correlations, one possibility would be to recouple ^15^N spins to ^13^C spins in a non-selective manner, i.e. N–CX rather than NCO or NCA. This method has been demonstrated previously, via a 3D-ZF-TEDOR-DARR experiment using broadband TEDOR rather than specific CP for ^15^N–^13^C polarisation transfer, simultaneously recoupling N–CO_i−1_, and N–Cα_i_ dipolar interactions, before a broadband ^13^C mixing period using DARR (Daviso et al. 2013). This method has the disadvantage of requiring that the entire protein ^13^C spectral width (≥ 175 ppm) be acquired in the indirect dimension of a 3D experiment, however, practically necessitating non-uniform sampling (NUS) or deliberate spectral aliasing in order to realise the acquisition timesaving over acquisition of separate NCOCX and NCACX spectra. Alternatively, one could exploit the ^13^C/^13^C homonuclear dipolar recoupling step to simultaneously recouple the Cα_i_–CO_i−1_ and Cα_i−1_–CO_i−1_ dipolar interactions after a specific ^15^N–^13^C CP transfer. However, many homonuclear dipolar recoupling sequences used for ^13^C/^13^C recoupling are not able to efficiently simultaneously recouple the smaller Cα_i_–CO_i−1_ dipolar interaction, as well as the Cα_i−1_–CO_i−1_ interaction, due to dipolar truncation and/or relayed transfers reducing the intensity of the comparatively long range Cα_i_–CO_i−1_ correlation (Bayro et al. 2009).

In this paper, we demonstrate that at moderate MAS frequencies (35 kHz), the mixed rotational and rotary resonance (MIRROR) homonuclear recoupling sequence may be incorporated into a 2D- or 3D-NCC correlation experiment to record simultaneously inter- and intra-residue MIRROR-NCOCA spectra by efficiently recoupling both the Cα_i−1_–CO_i−1_ and Cα_i_–CO_i−1_ dipolar interactions after a specific ^15^N–^13^C CP transfer step. The MIRROR sequence was chosen as it is a second-order, band-selective homonuclear recoupling sequence, that requires only low ^1^H rf amplitudes and has been shown to be efficient at moderate MAS frequencies (Scholz et al. 2008). First-order sequences have been utilized to band selectively recouple the Cα–CO dipolar interaction including band selective homonuclear CP (BSH-CP) (Shi et al. 2014; Chevelkov et al. 2013) and rotational resonance in a tilted rotating frame (R2TR) (Takegoshi et al. 1995; Detken et al. 2001a, b). Such sequences are however more susceptible to dipolar truncation than second order recoupling sequences supressing transfer through the longer range Cα_i_–CO_i−1_ interaction (Shi et al. 2014; Chevelkov et al. 2013). Broadband homonuclear recoupling sequences such as PDSD and DARR, which operate well at low magnetic fields and spinning frequencies were not chosen, as relayed transfers to sidechain ^13^C spins would potentially dilute the required spin polarisation transferred from the CO to the Cα. The same is true of broadband second-order sequences that operate efficiently at higher magnetic fields and spinning rates, such as PARIS (Weingarth et al. 2009), SHANGHAI (Hu et al. 2011) and PAR (Lewandowski et al. 2009).

The reintroduction of the second-order terms under the MIRROR condition relies on setting the amplitude of the proton rf irradiation (${\nu }_{1}^{H}$) to a multiple of the spinning speed (${\nu }_{r}$) plus or minus the isotropic chemical shift difference between the ^13^C nuclei (CO to Cα) to be recoupled (${\Delta \nu }_{iso}^{C}$),

1 $${\nu }_{1}^{H}=n{\nu }_{r}\pm {\Delta \nu }_{iso}^{C}.$$

As the recoupling condition is dependent on the isotropic chemical shift separation, the transfers are inherently selective. Band-selective transfers offer a number of advantages for backbone assignments, facilitating the directed transfer of magnetization along the protein backbone, whilst restricting the transfer of magnetization to the protein sidechains which would otherwise compromise signal intensity. These properties have previously been exploited in assignment schemes which exploit band-selective cross polarization (Chevelkov et al. 2013) and rotational resonance in the tilted rotating frame (Detken et al. 2001). At moderate MAS frequencies (40 kHz) the MIRROR condition has been shown to efficiently recouple the Cα–CO dipolar interaction to record ^13^C/^13^C correlation spectra in uniformly labelled proteins (Scholz et al. 2008). Several groups have utilised MIRROR to assist in characterization in a variety of systems (Cukkemane et al. 2012; Schuetz et al. 2010a, b; Wasmer et al. 2010). Here, we show that the efficiency of the MIRROR sequence is such that it may be used to recouple both the Cα_i−1_–CO_i−1_ and Cα_i_–CO_i−1_ dipolar interactions in one bidirectional transfer step. Furthermore, the polarization transfer to Cα_i_ and Cα_i−1_ spins is relatively uniform throughout the protein sequence, which we attribute to the fixed geometry of the peptide plane regardless of secondary structure. These transfer properties facilitate the reliable, robust observation of both inter- and intra-residue correlations. It is shown that the MIRROR sequence can be incorporated into a 3D-NCOCA experiment, and that the 3D-MIRROR-NCOCA alone can be used to straightforwardly assign the backbone resonances of a microcrystalline sample of the 56-residue model protein GB3, representing a 50% saving in terms of acquisition time over separate NCOCX and NCACX experiments. Moreover, since MIRROR uses relatively low-power rf fields, it can be combined with low-power CP and decoupling schemes permitting the construction of a low-power MIRROR-NCOCA experiment where recycle delays are limited by the T_1_ of the sample rather than the probe duty cycle. Together these properties allow for the efficient acquisition of a 3D data set which allows a complete backbone assignment to be made in a single experiment acquired within 24 h.

## Experimental section

### Sample preparation

Uniformly labelled ^13^C, ^15^N GB3 was expressed and purified using the following protocol. A plasmid encoding GB3 was transformed into *E. coli* BL21(DE3) cells, then grown at 37 °C in M9 minimal media (6 g/l Na_2_HPO_4,_ 3 g/l K_2_HPO_4_, 0.5 g/l NaCl, 1 mM MgSO_4_, 1 mM CaCl_2_) supplemented with 1 g/l ^15^NH_4_Cl, 2 g/l U–^13^C-glucose. Protein expression was induced at an OD_600_ of ~ 0.7, with 1 mM isopropyl ß-d-thiogalactoside at 18 °C for 16 h. Cells were pelleted and resuspended in PBS (137 mM NaCl, 2.7 mM KCl, 10 mM Na_2_HPO_4_, pH 7.4). The cells were then disrupted by sonication and insoluble material removed by centrifugation. The supernatant was heated to 80 °C for 10 min and the insoluble material removed by centrifugation. Ammonium sulphate was added to the supernatant to 60% saturation and allowed to equilibrate for 2 h, precipitant was again separated by centrifugation. The GB3 was subsequently precipitated in 90% ammonium sulphate. The pellet was dissolved in 25 mM Tris, pH 8, and desalted with a PD-10 gel filtration column. Anion exchange chromatography performed with 25 mM Tris, pH 8 (5 ml HiTrap Q HP), with GB3 eluting at ~ 0.25 M NaCl. Monomeric GB3 was isolated by gel-filtration (Sephadex 75) with 100 mM NaCl, 25 mM bis-tris, pH 6.5. Peak fractions were pooled and concentrated to ~ 30 mg/ml with Generon Vivaspin 5 kDa MWCO filters. Crystallisation was achieved by addition of 2-methyl-2,4-pentanediol to 60% v/v of the total sample volume and allowed to incubate at 0 °C for ~ 48 h. The sample was centrifuged into a 1.6/1.3 mm zirconia rotor. A silicone based glue was used to seal the rotor to maintain sample hydration.

### Solid-state NMR spectroscopy

**Fig. 1:**
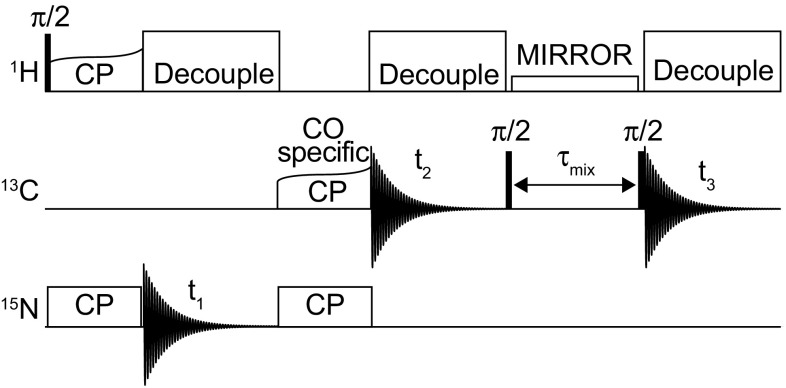
The 3D-MIRROR-NCOCA pulse sequence employed in this paper. 2D-NcoCA data were acquired with no CO evolution in the indirect dimension, t_2_. 1D-ncoCA spectra used for determining MIRROR conditions and CO/Cα transfer were acquired with no evolution in either the ^15^N (t_1_) or CO (t_2_) indirect dimensions. All other experimental details are reported in the materials and methods

Unless otherwise stated all measurements were conducted at 14.1 T on an Agilent DD2 600 MHz NMR spectrometer (Yarnton, UK) equipped with a 1.6 mm triple resonance magic-angle spinning probe. Samples were spun at 35 kHz, and the temperature regulated to 0 °C. For all triple resonance experiments the pulse sequence shown in Fig. 1 was employed. For ‘high-power’ ^1^H/^15^N CP the carrier frequencies were set to the centre of the ^1^H (~ 5 ppm) and ^15^N (~ 120 ppm) spectrum. Optimal ^1^H/^15^N cross-polarization was obtained with a 1.5 ms contact pulse with a ^1^H field of ~ 105 kHz and a ^15^N field of ~ 70 kHz. For ‘high-power’ ^15^N–^13^C CP the ^15^N carrier frequency was set to the middle of the amide region (120 ppm) and the ^13^C to the centre of the CO region (~ 172 ppm). The spin-lock fields were set to 5/2 times the spinning speed, 87.5 kHz, for ^15^N and 3/2 times the spinning speed, 52.5 kHz, for ^13^C. Maximal transfer was observed after 7 ms cross-polarization. During ^15^N/^13^C cross-polarization, 135 kHz continuous wave proton decoupling was applied. Under such conditions, magnetization transfer occurred exclusively from the ^15^N to the carbonyl carbons. Following transfer from the amide nitrogens to the carbonyls in the protein backbone, magnetization was transferred between carbon sites using a weak rf-field on the protons that satisfied either the dipolar assisted rotary resonance condition (${\nu }_{1}^{H}$ = $n\nu$_r_) or the MIRROR condition $\left( {\nu _{1}^{H} = n\nu _{r} \pm \Delta \nu _{{iso}}^{C} } \right)$), where ${\nu }_{1}^{H}$ is the proton rf-field amplitude, ν_r_ is the rotation frequency and ${\Delta \nu }_{iso}^{C}$ is the isotropic chemical shift difference of a chosen ^13^C spin-pair. During all evolution periods 120 kHz SPINAL proton decoupling was applied with phase flip angles of 10° and 5°. All π/2 pulses on ^1^H, ^13^C and ^15^N were set to at 2.4, 3.2, and 3.1 µs respectively.

For ‘low-power’ measurements, high-power decoupling was replaced with low-power TPPM decoupling (Kotecha et al. 2007) with a phase alternation of 20° and a pulse length of 55 µs. For ^1^H/^15^N and ^15^N/^13^C transfers low-power recoupling conditions were established with spin-lock fields of 62 kHz on ^1^H and 8 kHz on ^15^N for ^1^H/^15^N, and 25 kHz on ^15^N and 10 kHz on ^13^C for ^15^N/^13^C CP transfers. All ‘hard’ pulses on ^1^H, ^13^C and ^15^N were kept at 2.4, 3.2, and 3.1 µs respectively.

All 2D and 3D experiments were acquired with States-TPPI phase sensitive detection, employing a ^15^N spectral width of 5 kHz and 64 complex points and a C_CO_ spectral width of 2.5 kHz and 32 complex points. All spectra were externally referenced to the downfield resonance of adamantane at 40.48 ppm compared to DSS (Morcombe and Zilm 2003). Multidimensional data sets were processed in NMRPipe (Delaglio et al. 1995), prior to analysis and assignment in CCPN Analysis 2.4 (Vranken et al. 2005; Stevens et al. 2011). All processing parameters are given in the corresponding figure legends.

## Results and discussion

**Fig. 2:**
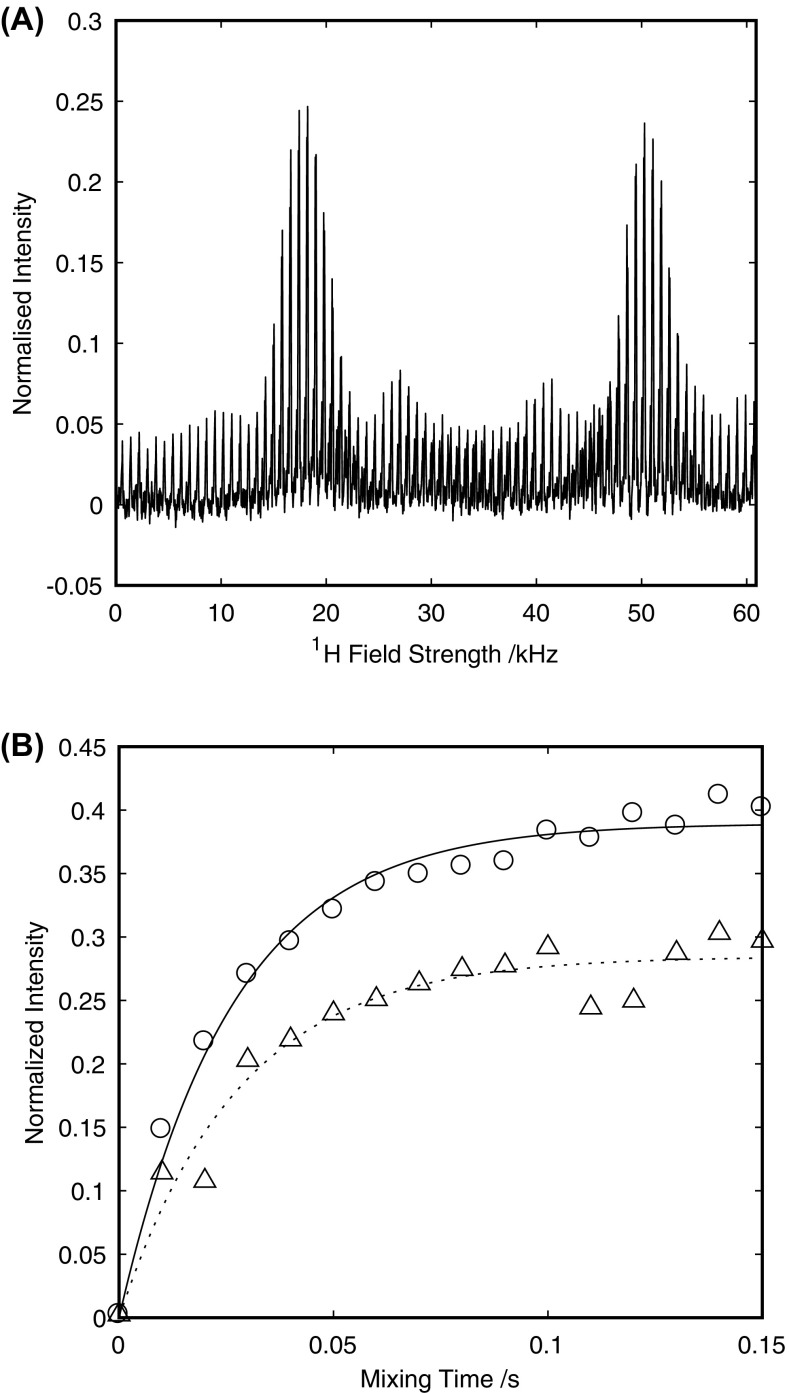
Signal from Cα region of a 1D-ncoCA spectrum of a microcrystalline GB3 following a 100 ms period of MIRROR recoupling as a function of the MIRROR recoupling rf-field (**a**). Build-up of Cα magnetization in a 1D-ncoCA spectrum of microcrystalline GB3 as a function of MIRROR recoupling period for the recoupling condition at 18.5 kHz (solid line with open circle) and 50 kHz (dotted line with open triangle) (**b**). The amplitudes in A and B are normalised to the integrated intensity of the carbonyl region in the absence of any MIRROR recoupling. Data acquired at 14.1 T with 35 kHz MAS

Optimal recoupling under the MIRROR condition occurs when the proton rf amplitude fulfils the condition ${\nu }_{1}^{H}=n{\nu }_{r}\pm {\Delta \nu }_{iso}^{C}$. Based on the isotropic chemical shifts obtained from the BMRB, (Ulrich et al. 2008) the average frequency separation, $\Delta \overline{\nu } _{{iso}}^{C}$, between the CO and Cα at 14.1 T, the field employed here, is 18.75 kHz. This gives rise to theoretical MIRROR conditions at proton rf amplitudes corresponding to nutation frequencies of 16.25, 18.75, 51.25 and 53.75 kHz. To identify conditions under which optimal transfer occurs experimentally, the Cα signal intensity obtained after 100 ms MIRROR mixing was plotted as a function of the MIRROR recoupling field (Fig. 2a). Transfer occurs at two conditions approximately 5 kHz wide that are centred at 18.75 and 50.5 kHz in good agreement with the predicted values $\left( {\nu _{1}^{H} = n\nu _{r} \pm \Delta \nu _{{iso}}^{C} } \right)$. The absence of any transfer at the n = 1 DARR condition (35 kHz) should be noted, highlighting the poor efficiency of this technique at moderate spinning frequencies. Comparison of the build-up of magnetization at these two matching conditions (Fig. 2b) shows that maximal transfer occurs with a ^1^H rf amplitude of 18.75 kHz, where transfer occurs under the n = 0 $\left( {\nu _{1}^{H} = + \Delta \nu _{{iso}}^{C} } \right)$ and n = 1 $\left({\nu }_{1}^{H}={\nu }_{r}-{\Delta \nu }_{iso}^{C}\right)$ resonance conditions. Although the superimposition of the two matching conditions leads to efficient transfer of magnetization, through the judicious choice of spinning speed the width of the matching condition can be tailored by varying the separation between the n = 0 $({\nu }_{1}^{H}=+{\Delta \nu }_{iso}^{C})$ and n = 1$\left({\nu }_{1}^{H}={\nu }_{r}-{\Delta \nu }_{iso}^{C}\right)$ conditions. Transfer under the 50.5 kHz matching condition results in a 25% reduction in the total amount of magnetization transferred from the CO to the Cα, mirroring earlier reports that showed maximal transfer efficiencies at the lower power matching conditions (Scholz et al. 2008). When normalised against the total intensity of the carbonyl region approximately 40% of the magnetization is transferred from the CO region to the Cα region of the spectrum, with the bulk of the magnetization transferred within the first 50 ms of recoupling. The extent of transfer is comparable to other band-selective transfers including band-selective cross-polarization albeit with significantly reduced rf ampliitudes (Chevelkov et al. 2013).

**Fig. 3:**
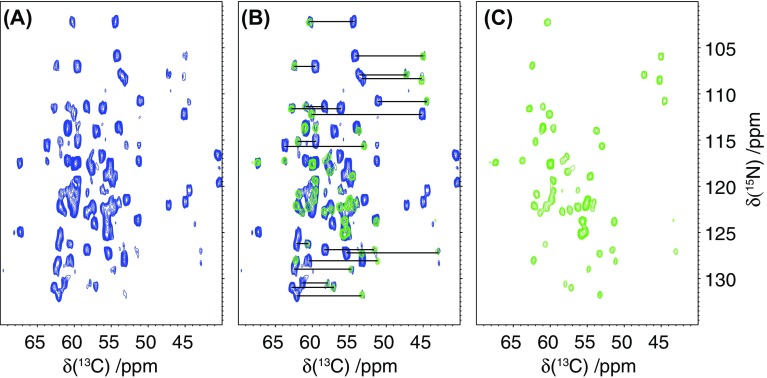
2D-NcoCA spectrum of microcrystalline GB3 using 100 ms MIRROR transfer from transfer of magnetization from the CO to Cα (**a**, blue), superimposition of the NcoCA (blue) with the NCA spectrum (green) (**b**), and the NCA spectrum (**c**, green). Tie lines in (**b**) highlight the presence of connectivities between intense peaks arising from the NCOCA_(i−1)_ and the weaker peaks arising from the NCOCA_(i)_ transfer. Data acquired at 14.1 T with 35 kHz MAS

2D-NcoCA data (Fig. 3a) demonstrate that efficient transfer is observed across the Cα region with resonances from 40 to 70 ppm exhibiting similar recoupling efficiencies. This suggests that at 14.1 T B_0_ field strength and 35 kHz spinning speed, the MIRROR recoupling bandwidth is sufficient to actively recouple the entire CO/Cα envelopes, even in the absence of any modulation of the MIRROR recoupling field. As expected, and in contrast to DARR experiments conducted at lower spinning speeds, little of the magnetization is relayed out to the sidechains following transfer of magnetization from the CO to Cα. Indeed, the resonances not attributable to Cα arise from correlations with ^13^C sites on amide sidechains. These exhibit a similar chemical shift dispersion and strong dipolar couplings to CO groups. The selective nature of the transfer limits the diffusion of magnetization throughout the protein leading to enhanced intensity in the Cα region of the spectrum.

To assess the utility of the technique at higher field where the variation in ${\Delta \nu }_{iso}^{C}$will increase with field and the recoupling bandwidth may become comparable to the chemical shift distribution, NcoCA data were acquired at 20 T (see Supplementary Fig. S1). At the higher field with 50 kHz MAS, transfer is still observed at the n = 0 $\left( {\nu _{1}^{H} = + \Delta \nu _{{iso}}^{C} } \right)$ and n = 1$\left({\nu }_{1}^{H}={\nu }_{r}-{\Delta \nu }_{iso}^{C}\right)$ condition, although the overall signal sensitivity is lower due to the smaller sample volumes available in the smaller diameter rotors. Intensity is observed from 70 to 40 pm, indicating that transfer occurs quite uniformly across the Cα region, even at the high magnetic field, and without the need to resort to amplitude or phase modulation of the MIRROR recoupling rf-field (Scholz et al. 2008; Wittmann et al. 2014).

**Fig. 4:**
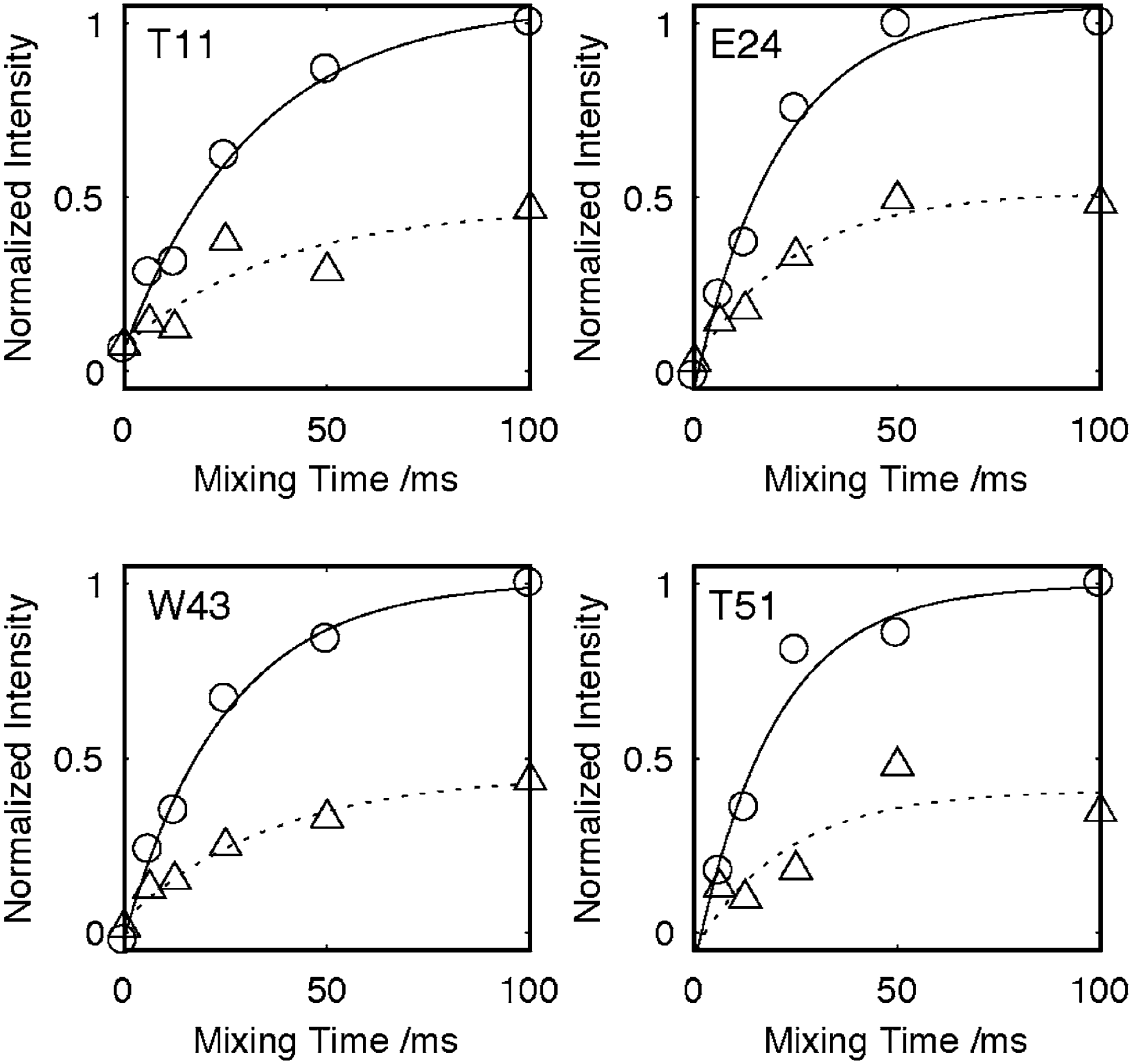
Plots showing the build-up of magnetization following transfer from CO_(i-1_) to Cα_(i-1)_ (solid line with open circle) and CO_(i-1)_ to Cα_(i)_ (dotted line with open triangle) for selected resonances. Data integrated from the corresponding 2D-NcoCA spectra of a microcrystalline preparation of GB3. All data normalised to the maximum intensity of the Cα_(i-1)_, and fitted to an exponential build-up. Data acquired at 14.1 T with 35 kHz MAS

Closer inspection of the 2D-NcoCA spectrum in Fig. 3a reveals that in addition to the intense resonances arising from the transfer of magnetization from N to CO_(i−1)_ to Cα_(i−1)_ weaker correlations whose intensity is approximately 50% of the intensity of the NCOCA_(i−1)_ are also present for each of the nitrogen sites. Comparison of this data with the corresponding NCA spectrum (Fig. 3b, c) reveals that these weaker resonances correspond to the transfer of magnetization of N to CO_(i−1)_ to Cα_(i)_. Typically at lower spinning speeds using broad-banded transfers we have not systematically observed these long range correlations, presumably due to dipolar truncation effects and loss of intensity as magnetization is transferred out to the amino acid side chains. In this instance though it is apparent that by band selectively recoupling the CO to the Cα region of the spectra correlations are observed to both the adjacent and more proximal Cα’s. Given the fixed geometry surrounding the peptide bond, the distances between the CO_(i−1)_ and the Cα_(i−1)_ and Cα_(i)_ remain constant at 1.52 and 2.43 Å respectively, irrespective of the backbone conformation of the protein. Given this fixed geometry, it is expected that the transfer of magnetization from the CO_(i−1)_ to the Cα_(i−1)_ and Cα_(i)_ would be similar for all sites within the proteins, something that is qualitatively apparent in the 2D-NcoCA spectrum of GB3 (Fig. 3a, b) where in well resolved regions each nitrogen resonance possesses pairs of Cα resonances irrespective of its location within the protein. A quantitative analysis of the magnetization transfer (Fig. 4, Supplementary Table S1) from the CO to the two Cα sites reveals that the rate of transfer to the two sites is similar. Time constants of between 20 and 40 ms are observed when exchange is modelled as an exponential process. This suggests that the observed difference in resonance intensities is not due to differences in transfer rates, but rather to differences in the extent to which magnetization is transferred. The more distant Cα_(i)_ site typically exhibits an intensity of 40–60% of that observed for the direct transfer to the Cα_(i−1)_ site (Fig. 3, Supplementary Table S1).

**Fig. 5:**
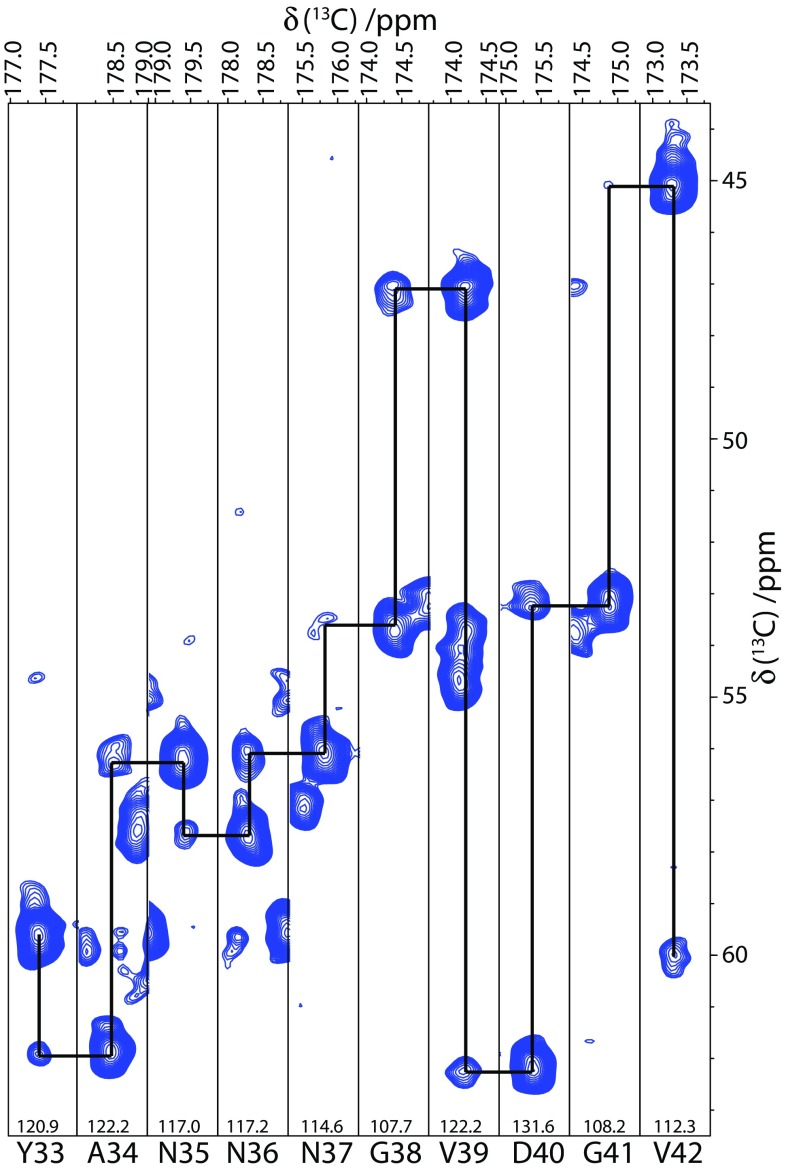
Strip plot of low-power 3D-NCOCA experiment for connectivities between residues Y33 to V42 with tracing through backbone. Chemical shift for each ^15^N plane is given at the bottom of each slice. Large peaks in each nitrogen dimension are the CO_(i−1)_ to Cα_(i−1)_, while the smaller peak is the CO_(i−1)_ to Cα_(i)_ correlation

In the context of a 2D-NcoCA experiment, the presence of the coupling from the CO_(i−1)_ to the Cα_(i)_ and Cα_(i−1)_ sites allows nitrogens in the peptide backbone to be simultaneously correlated to the (i) and (i–1) residues in a single experiment. The presence of additional peaks in the NCOCA spectrum has previously been observed when employing broad-banded homonuclear recoupling sequence such as PDSD and in such instances viewed unfavourably as it can lead to increased spectral crowding (Chevelkov et al. 2013). This spectral crowding can in part be overcome through the acquisition of 3D-NCOCA spectra (Supplementary Fig. S3). Importantly though, the MIRROR transfer results in systematic and efficient transfer from the CO_(i−1)_ to Cα_(i)_ and Cα_(i−1)_ sites with little/no coupling to other sites. This simplifies the analysis of the data as the resulting Cα strip plots contains only two resonances, the stronger showing the correlation between N_(i)_ and Cα_(i−1)_ and the weaker that between N_(i)_ and Cα_(i)_ (Supplementary Fig. S3 and Fig. 5). This facilitates the assignment process as all the connectivities necessary for a backbone assignment are present in a single data set. For those familiar with solution NMR protein backbone assignments, the resulting spectrum can be thought of as analogous to the liquid state HNCA (Kay et al. 1990; Farmer et al. 1992; Grzesiek and Bax 1992), albeit with an inversion of the relative intensities of the Cα resonances of the current and preceding amino acid. The spectra can therefore be interpreted in a similar manner to solution-state HNCA data, ‘walking’ through the Cα slices to sequentially assign the protein backbone.

The low rf amplitudes employed for MIRROR recoupling offer significant advantages for the analysis of protein samples, since rf-induced sample heating is greatly limited. The use of weak rf-fields permits a reduction in recycle delay, whose minimum value is normally limited by the duty cycle of the probe/spectrometer under high-power conditions. Furthermore, MIRROR recoupling and the assignment scheme highlighted above is compatible with the low-power decoupling and CP schemes (Kotecha et al. 2007; Vijayan et al. 2009; Ernst et al. 2003) and PACC schemes (Wickramasinghe et al. 2008; Ishii et al. 2001).

To demonstrate the potential savings in acquisition time we have incorporated the MIRROR recoupling into an NCOCA experiment where all polarization transfer and decoupling steps have been replaced by low-power equivalents (Fig. 5), allowing the recycle time to be reduced from 2.5 s to that which is optimal for the T_1_ of the sample (700 ms). During all evolution times, SPINAL decoupling (Fung et al. 2000) was replaced by low-power TPPM (Kotecha et al. 2007), allowing a reduction of ^1^H rf amplitudes from 135 to 8.75 kHz. Low-power CP conditions can also be identified by arraying both ^1^H and ^15^N field strengths during the CP period to locate a suitable low-power condition, with efficient transfer from ^1^H to ^15^N achieved using double quantum CP at the n = 2 condition (Meier 1992; Laage et al. 2009; Demers et al. 2010), with a ^1^H spin-lock field of 62 kHz and a ^15^N of 8 kHz, whilst optimal transfer from ^15^N to ^13^C obtained at the n = 1 double quantum CP condition with a ^13^C spin-lock field of 10 kHz and a ^15^N field of 25 kHz. A comparison of the peak intensities of these conditions with the high-power variants is given in Fig. S2. As is apparent, the replacement of polarization transfer steps with low-power equivalents has little effect on the overall resolution and sensitivity of the overall experiment, even at these moderate spinning frequencies. The only noticeable difference between the low-power and high-power variants is the absence of intensity between 38 and 42 ppm which we attribute to the sidechains of the acidic amino acids.

The reduction in recycle time allowed a single 3D-NCOCA experiment, providing the complete backbone assignment of the 56 residue protein GB3, to be performed in as little as 12.5 h. This may be compared with 45 h for the equivalent high-power experiment. A comparison of the data quality shown in Supplementary Fig. S3. Further time saving may be made through the use of paramagnetic dopants to accelerate T_1_ relaxation, allowing a further reduction in recycle time.

## Conclusion

In summary, we demonstrate that MIRROR recoupling enables the bidirectional transfer of magnetization from the CO site to the adjacent Cα, and to the Cα of the preceding amino acid. In the context of a 3D-NCOCA experiment this doubles the information content providing correlations from CO(_i−1_) to both Cα_(i−1)_ and Cα_(i)_. The presence of these two correlations permits the sequential assignment of the protein backbone without the need for conducting multiple 3D experiments. Furthermore, the low rf amplitudes required for the most efficient MIRROR recoupling conditions enable the construction of ‘low-power’ experiments facilitating further reductions in the data acquisition time.

## Electronic supplementary material

Below is the link to the electronic supplementary material.

Supplementary material 1 (PDF 564 KB)
