# Supplementary material for: Bidirectional band-selective magnetization transfer along the protein backbone doubles the information content of solid-state NMR correlation experiments
Source: J Biomol NMR. 2017 Nov 8;69(4):197–205. doi: 10.1007/s10858-017-0147-0 (PMC5736786; doi:10.1007/s10858-017-0147-0)
Supplement: Supplementary file 1 — Supplementary material 1 (PDF 564 KB) [file 10858_2017_147_MOESM1_ESM.pdf]

Bidirectional Band-Selective Magnetization Transfer Along the Protein Backbone Doubles the Information Content of Solid-State NMR Correlation Experiments.

M.M. Jolly[1], J.A. Jarvis[1], M. Carravetta[2], M.H. Levitt[2], P.T.F. Williamson[1†]

[1]Centre for Biological Sciences, University of Southampton, Southampton,

United Kingdom, SO17 1BJ

[2]School of Chemistry, University of Southampton, Southampton,

United Kingdom, SO17 1BJ

[†] *Corresponding author*

Supplementary Information

Contents:

1. Supplementary Figure 1: Comparison of bandwidths at high magnetic fields.
2. Supplementary Figure 2: Efficiency of 'low-power' sequences.
3. Supplementary Figure 3: Comparison of high-power and low-power data collection schemes.
4. Supplementary Table 1: Rates of transfer from CO to $Ca_{(i)}$ and $Ca_{(i-1)}$ together with the ratio between the final peak intensities.

Supplementary Figure 1: Comparison of bandwidths at high magnetic fields.

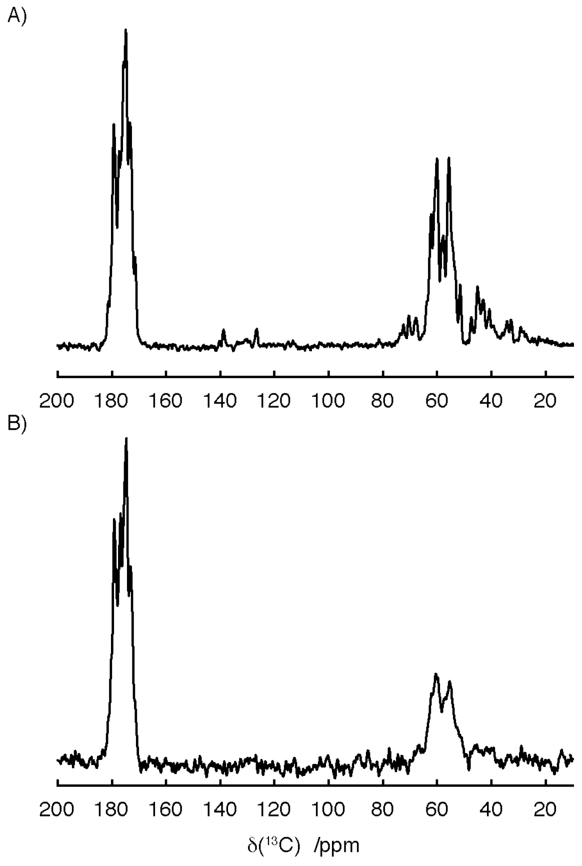

Fig. S1. Comparison of recoupling bandwidth at 14.1 T and 20 T, 600 and 850 MHz proton Larmor frequency respectively. 1D-ncoCA spectrum of GB3 acquired at 35 kHz with 18.75 kHz MIRROR recoupling field, the n = 0, $+\Delta\nu_{iso}^{C}$ recoupling condition at 14.1T (A). 1D-ncoCA spectrum of GB3 acquired at 50 kHz with 26.5 kHz MIRROR recoupling field, the n = 0, $+\Delta\nu_{iso}^{C}$ recoupling condition at 20 T (A). Data acquired with a 1.3 mm TR-MAS probe on a Bruker AVANCE-II 850 MHz spectrometer. The overall sensitivity of these experiments was significantly attenuated by the small sample volumes in the 1.3 mm probe, where proton detected experiments would be more

routine. Despite this, these data highlight that even at the higher fields, the recoupling efficiency is sufficiently broad to allow efficient recoupling of the entire CO/CA envelopes.

Supplementary Figure 2: Efficiency of ‘low-power’ sequences.

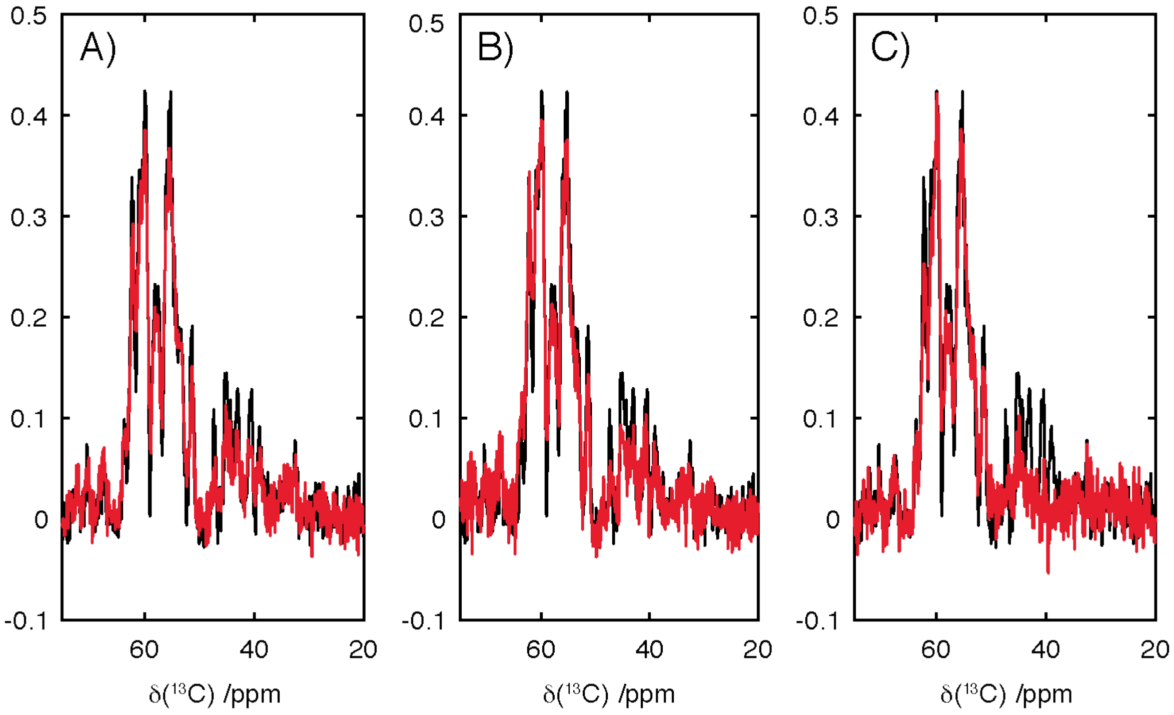

Fig. S2. Comparison of transfer efficiency upon incorporating low power decoupling and CP conditions. High power NcoCA (black, A/B/C), acquired with ^{1}H and ^{15}N spin lock fields of 122 and 87.5 kHz respectively during ^{1}H/^{15}N and ^{15}N and ^{13}C spin lock fields of 87.5 and 52.5 kHz respectively for ^{15}N/^{13}C CP. During acquisition 135 kHz SPINAL decoupling was applied. Effect of sequentially replacing high-power SPINAL decoupling with low power TPPM (8.75 kHz rf field) (red, A), high-power ^{1}H/^{15}N CP with a low-power DQ-CP condition (red, B), and a low power ^{13}C/^{15}N DQ-CP transfer (red, C). Data normalised to maximum intensity observed under high-power conditions.

Supplementary Figure 3: Comparison of high-power and low-power data collection schemes.

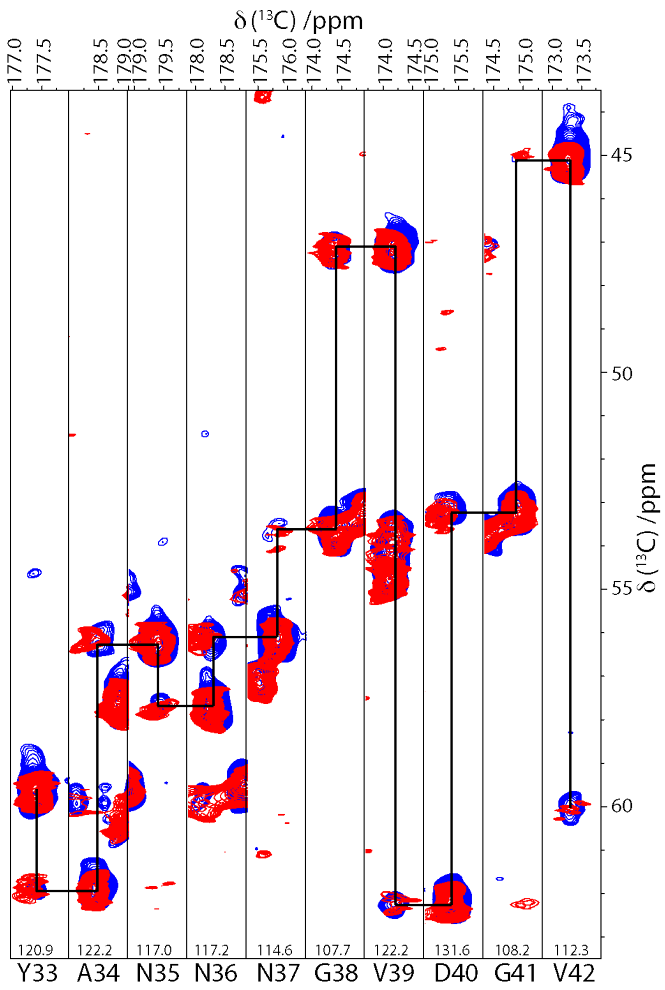

Fig. S3. Overlay of a high-power 3D-NCOCA MIRROR experiment (red), with that of a low-power 3D-NCOCA MIRROR experiment (blue). The chemical shift for the ^{15}N plane is given at the bottom of each slice. 3D experiments were acquired with States-TPPI phase sensitive detection, employing a ^{15}N spectral width of 5 kHz and 64 complex points and a $^{13}C_{CO}$ spectral width of 2.5 kHz and 32 complex points. For both the direct and indirect ^{13}C dimensions the data was processed with a Lorentz-to-Gauss window function, with inverse exponential width of 10 Hz and a Gaussian broaden width of 40 Hz. In the indirect ^{13}C dimension the data was linear predicted to 128 complex

data points. For the indirect ^{15}N dimension the data was processed with a Lorentz-to-Gauss window function, with inverse exponential width of 20 Hz and a Gaussian broaden width of 40 Hz.

Supplementary Table 1: Rates of transfer from CO to $C\alpha_{(i)}$ and $C\alpha_{(i-1)}$ together with the ratio between the final peak intensities.

Residue	NCA /sec	NcoCA /sec	$I_{NCA/NcoCA}$
2GlnCa*	0.035	0.043	1.53
3TyrCa	0.040	0.069	0.68
4LysCa*	0.040	0.039	0.68
5LeuCa	0.037	0.041	0.38
6ValCa*	0.030	0.048	0.77
7IleCa	0.041	0.038	0.39
8AsnCa	0.014	0.043	0.74
9GlyCa	0.019	0.035	0.44
10LysCa*	0.043	0.042	0.69
11ThrCa	0.031	0.037	0.34
12LeuCa	0.026	0.038	0.34
13LysCa*	0.031	0.033	1.03
14GlyCa	0.025	0.036	0.45
15GluCa	0.026	0.033	0.80
16ThrCa*	0.041	0.040	1.03
17ThrCa*	0.025	0.036	0.52
18ThrCa	0.049	0.040	0.33
19LysCa*	0.028	0.038	1.12
20AlaCa*	0.027	0.033	0.79
21ValCa	0.014	0.038	0.16
22AspCa	0.035	0.054	0.46
23AlaCa	0.037	0.041	0.60
24GluCa	0.028	0.047	0.38
25ThrCa*	0.027	0.039	0.95
26AlaCa*	0.045	0.033	0.48
27GluCa*	0.045	0.046	0.98
28LysCa*	0.045	0.043	1.20
29AlaCa*	0.037	0.046	0.81
30PheCa*	0.036	0.038	0.31
31LysCa*	0.040	0.051	1.22
32GlnCa*	0.042	0.055	1.10
33TyrCa	0.042	0.063	0.36
34AlaCa	0.032	0.036	1.06
35AsnCa	0.033	0.064	0.75
36AspCa	0.026	0.051	0.42
37AsnCa*	0.024	0.033	0.58

38GlyCa	0.018	0.055	0.64
39ValCa*	0.059	0.037	1.25
40AspCa	0.023	0.036	0.67
41GlyCa	0.031	0.048	0.27
42ValCa	0.040	0.031	0.50
43TrpCa	0.035	0.040	0.56
44ThrCa	0.019	0.047	0.79
45TyrCa	0.045	0.043	0.91
46AspCa	0.059	0.048	0.32
47AspCa*	0.038	0.035	1.30
48AlaCa*	0.025	0.050	0.81
49ThrCa	0.038	0.033	0.40
50LysCa	0.023	0.053	0.47
51ThrCa	0.034	0.053	0.48
52PheCa	0.013	0.037	0.86
53ThrCa*	0.041	0.054	2.61
54ValCa	0.025	0.042	0.75
55ThrCa	0.023	0.036	0.56
56GluCa	0.016	0.043	0.53

Table S1. Analysis of rate of transfer from the CO to the $CA_{(i)}$ and $CA_{(i-1)}$ resonances and ratio of the two intensities for assigned resonances in GB3. *Denotes regions with spectral overlap resulting in anomalous ratios.
